# Development and External Validation of a Multivariable Model to Predict Early Minimal Symptom Expression Response in Adult Generalized Myasthenia Gravis Patients Treated With Efgartigimod

**DOI:** 10.1002/cns.70746

**Published:** 2026-01-12

**Authors:** Yufang Yang, Tao Liang, Mingming Zhao, Hongxia Yang, Zhilan Zhao, Lu Yu, Linlin Yan, Siyuan Li, Peng Zhang, Guoyan Qi, Jian Yin, Zucai Xu, Zhong Luo

**Affiliations:** ^1^ Department of Neurology Affiliated Hospital of Zunyi Medical University Zunyi China; ^2^ Key Laboratory of Brain Function and Brain Disease Prevention and Treatment of Guizhou Province (Qiankehe Foundation‐ZSYS(2025)030) Zunyi China; ^3^ Department of Neurology Beijing Hospital, National Center of Gerontology, Institute of Geriatric Medicine, Chinese Academy of Medical Sciences Beijing China; ^4^ Center for the Diagnosis and Treatment of Myasthenia Gravis Shijiazhuang People's Hospital Shijiazhuang China; ^5^ Department of Neurology Second Affiliated Hospital of Guizhou University of Traditional Chinese Medicine Guiyang China; ^6^ Department of Neurology Guizhou Provincial People's Hospital Guiyang China

**Keywords:** generalized Myasthenia Gravis, minimal symptom expression, nomogram, predictive model

## Abstract

**Background:**

Efgartigimod, a neonatal Fc receptor (FcRn) blocker, is approved for generalized Myasthenia Gravis (gMG), but predictors of early response are unclear. Using minimal symptom expression (MSE) as the therapeutic target, we developed and externally validated a clinical model to predict early MSE response after starting efgartigimod. This efgartigimod‐tailored model estimates the baseline probability of achieving early MSE and may assist in individualized treatment selection at therapy initiation.

**Methods:**

We retrospectively analyzed 118 adults with gMG treated at five tertiary centers in China (efgartigimod 10 mg/kg IV weekly ×4). MSE was defined as Myasthenia Gravis–Activities of Daily Living (MG‐ADL) ≤ 1 sustained ≥ 4 weeks; early MSE response was achievement within 4 weeks of initiation. Patients were split into a derivation cohort (*n* = 64; three centers) and an external validation cohort (*n* = 54; two centers). Candidate predictors included demographics, baseline severity, and laboratory indices. Variables associated with early MSE in univariable analyses entered multivariable logistic regression to construct a nomogram. Discrimination (AUC‐ROC), calibration (curves and Spiegelhalter *Z*‐test), and clinical utility (decision curve analysis, DCA) were assessed, with bootstrap internal validation.

**Results:**

Early MSE response occurred in 26/64 derivation and 22/54 validation patients. Lower bulbar MG‐ADL (OR 0.633, *p* = 0.040), higher FVC% (OR 1.042, *p* = 0.048), and lower IgG (OR 0.795, *p* = 0.036) independently predicted early MSE response. The nomogram showed strong discrimination—derivation AUC 0.869 (95% CI 0.797–0.941), bootstrap AUC 0.880 (0.806–0.954), and external AUC 0.839 (0.760–0.919)—and good calibration (Spiegelhalter *Z*: 1.03, *p* = 0.303; 0.94, *p* = 0.347; 1.17, *p* = 0.242). DCA indicated net benefit across thresholds 0.05–0.82, with validation curves mirroring derivation.

**Conclusions:**

A three‐factor nomogram (bulbar MG‐ADL, FVC%, and serum IgG) provides an efgartigimod‐specific baseline estimate of early sustained‐MSE response probability, which may help neurologists select appropriate candidates, counsel expected benefit, and tailor follow‐up intensity or alternative escalation strategies.

**Trail Registration:**

Chinese Clinical Trial Registry (ChiCTR2500101971)

## Graphical abstract

A multicenter adult gMG cohort from five tertiary centers in China (*N* = 118) received efgartigimod 10 mg/kg IV weekly × 4. Early minimal symptom expression (MSE) response was defined as MG‐ADL ≤ 1 sustained ≥ 4 weeks and assessed within 4 weeks. A three‐factor nomogram (bulbar MG‐ADL subscore, FVC% predicted, serum IgG) was developed in the derivation cohort (*n* = 64), internally validated by bootstrap, and externally validated (*n* = 54). The model showed strong discrimination (ROC/AUC), good calibration, and meaningful net clinical benefit on decision‐curve analysis, enabling individualized prediction of early MSE response to efgartigimod.

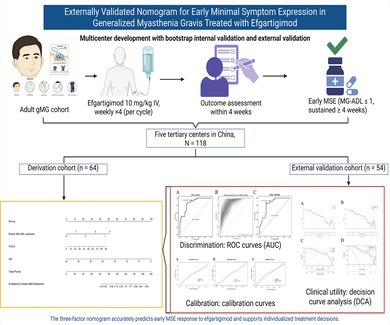

**Abbreviations**
Ab: antibody
AChR: acetylcholine receptor
AChR‐Ab: acetylcholine receptor autoantibody
AUC: area under the ROC curve
CI: confidence interval
DCA: decision curve analysis
FcRn: neonatal Fc receptor
FVC: forced vital capacity (absolute)
FVC%: percent predicted forced vital capacity
gMG: generalized Myasthenia Gravis
HBV: hepatitis B virus
HCV: hepatitis C virus
HIV: human immunodeficiency virus
IMC: impending myasthenic crisis
IV: intravenous
IVIg: intravenous immunoglobulin
LRP4: low‐density lipoprotein receptor–related protein 4
MC: myasthenic crisis
MG: Myasthenia Gravis
MG‐ADL: Myasthenia Gravis activities of daily living
MGFA: Myasthenia Gravis Foundation of America
MuSK: muscle‐specific tyrosine kinase
MuSK‐Ab: muscle‐specific tyrosine kinase autoantibody
NMJ: neuromuscular junction
NSIST: non‐steroidal immunosuppressants
OR: odds ratio
PE: plasma exchange
PRO: patient‐reported outcome
QMG: quantitative Myasthenia Gravis (score)
ROC: receiver operating characteristic
RyR: ryanodine receptor
RyR‐Ab: ryanodine receptor autoantibody
SD: standard deviation
TB: tuberculosis
TRIPOD: Transparent Reporting of a multivariable prediction model for Individual Prognosis or Diagnosis

## 1 Introduction

Myasthenia gravis (MG) is an autoimmune disorder of neuromuscular transmission at the neuromuscular junction (NMJ), predominantly mediated by immunoglobulin G (IgG) antibodies [1]. Antibodies against the acetylcholine receptor (AChR) are the most common pathogenic antibodies. Additionally, antibodies directed at other postsynaptic components—including muscle‐specific tyrosine kinase (MuSK), low‐density lipoprotein receptor‐related protein 4 (LRP4), ryanodine receptor (RyR), and titin—have been implicated in MG pathogenesis by disrupting AChR clustering, altering AChR function, and impairing NMJ signaling [2]. Up to 85% of patients present with generalized Myasthenia Gravis (gMG), and approximately two‐thirds of patients initially diagnosed with ocular MG develop gMG within 1–3 years [3]. Current gMG therapies include cholinesterase inhibitors, corticosteroids, non‐steroidal immunosuppressants, intravenous immunoglobulin (IVIg), plasma exchange, and thymectomy. However, corticosteroids and non‐steroidal immunosuppressants exert broad immunosuppression without selectively targeting IgG autoantibodies central to gMG pathogenesis, often yielding insufficient symptom control and significant adverse effects [4].

Efgartigimod is a human IgG1 Fc fragment and a natural ligand of FcRn. Compared with endogenous IgG, it exhibits higher affinity for FcRn while preserving pH‐dependent binding, thereby outcompeting endogenous IgG for FcRn, reducing IgG recycling, and increasing IgG degradation [1]. In the Phase 3 ADAPT trial, selective IgG reduction via FcRn blockade with efgartigimod was a safe, effective, and well‐tolerated add‐on therapy for gMG, with 40% of AChR‐positive patients achieving MSE during the first treatment cycle [4]. Multiple real‐world studies, primarily in AChR‐Ab positive gMG, report that 10.4%–40% of patients achieve MSE during follow‐up [5, 6, 7, 8, 9, 10]; nevertheless, 20%–49.1% of patients have an inadequate response, and rates of myasthenic crisis exceed those reported in clinical trials [11]. These findings underscore substantial heterogeneity in treatment response.

Despite confirmed overall efficacy, no clinical tool currently predicts which gMG patients are most likely to benefit from efgartigimod. The absence of a reliable predictive model increases uncertainty in clinical decision‐making and may expose some patients to unnecessary treatment and economic burden. Developing and validating a predictive model can provide risk stratification, enabling clinicians to identify early those patients most likely to respond to efgartigimod and to optimize individualized therapeutic strategies. Intended users include neurologists, immunologists, and multidisciplinary teams guiding treatment decisions. In China, regional disparities in health‐care resources, delays in diagnosis, variable drug accessibility, and the high costs of biologics—particularly burdensome for low‐income patients—further justify a response‐prediction model that could reduce unnecessary expenditures and improve treatment equity.

This study aimed to develop and externally validate a multivariable prediction model estimating the probability of an early MSE response in adult gMG patients receiving efgartigimod. We performed a multicenter retrospective cohort study, integrated demographic and baseline clinical characteristics to construct a nomogram, and evaluated its discrimination, calibration, and clinical utility.

## 2 Methods

### 2.1 Study Population

This retrospective, multicenter, observational cohort enrolled 203 gMG patients who received at least one cycle of adjunct efgartigimod therapy between October 2023 and July 2025 at five tertiary medical centers in China. Generalized MG was diagnosed by neuromuscular specialists at each participating center based on typical fluctuating fatigable weakness with objective clinical findings, supported by MG‐related autoantibody testing and/or electrophysiological evidence (repetitive nerve stimulation decrement or single‐fiber EMG increased jitter), with alternative diagnoses excluded. Generalized status was defined as involvement beyond extraocular muscles (MGFA clinical class II–V); patients with purely ocular MG (MGFA class I) were not eligible.

### 2.2 Inclusion and Exclusion Criteria

Inclusion criteria: (1) age ≥ 18 years with a confirmed diagnosis of gMG; (2) seropositivity for one or more of AChR‐Ab, MuSK‐Ab, LRP4‐Ab, titin‐Ab, or RyR‐Ab; (3) Myasthenia Gravis Foundation of America (MGFA) clinical classification II–V; (4) MG‐ADL score ≥ 6; (5) efgartigimod 10 mg/kg intravenously once weekly for four consecutive weeks per cycle, with ≥ 1 cycle completed; and (6) ≥ 6 months from efgartigimod initiation to last follow‐up.

Exclusion criteria: (1) active hepatitis B, positive hepatitis C antibody, positive HIV antibody, or active pulmonary tuberculosis; (2) pregnancy, lactation, or intention to conceive during the study period; (3) investigator‐determined unsuitability (e.g., severe psychiatric disorders); and (4) incomplete baseline clinical data.

**FIGURE 1:**
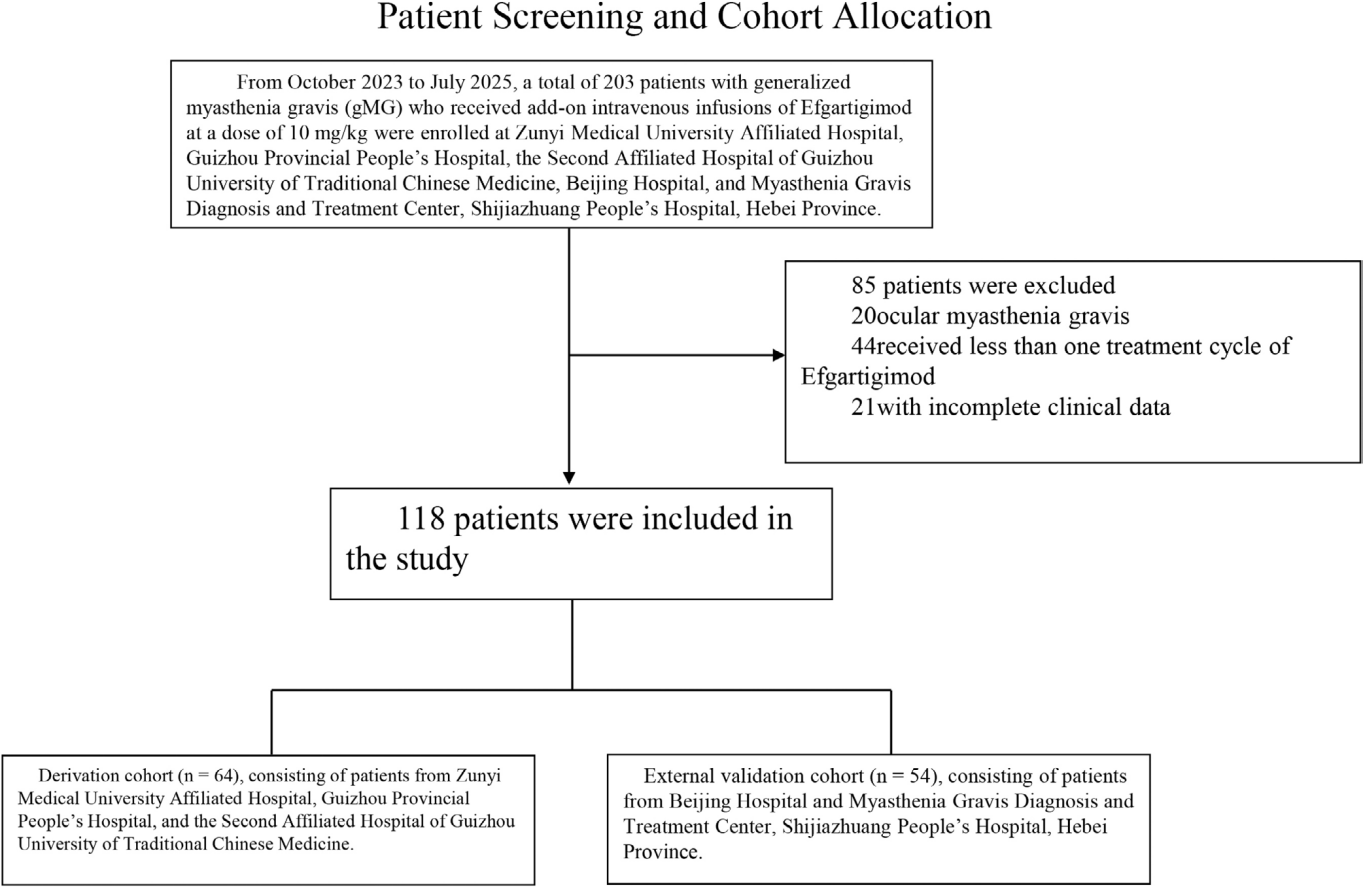
Patient screening and cohort allocation. From October 2023 to July 2025, 203 patients with generalized Myasthenia Gravis (gMG) who received at least one cycle of adjunct efgartigimod (10 mg/kg IV weekly ×4) were screened across five tertiary centers. After excluding 20 patients with ocular MG, 44 who received < 1 treatment cycle, and 21 with incomplete baseline data, 118 patients were included and split by center into a derivation cohort (*n* = 64) and an external validation cohort (*n* = 54).

Efgartigimod was used as adjunct therapy in routine practice. Concomitant MG medications at treatment initiation (pyridostigmine, corticosteroids, and non‐steroidal immunosuppressants) as well as prior rescue therapy exposure (IVIg/plasma exchange) were recorded at baseline and handled as clinical covariates (candidate predictors and subgroup strata); no protocolized washout or mandated dose standardization was required in this retrospective cohort. Among screened patients excluded for incomplete baseline information (Figure 1), the most commonly missing baseline variables were respiratory function measures (FVC and/or FVC%) and serum IgG. Therefore, the final model development and validation were conducted as a complete‐case analysis, and no imputation was performed.

Ethics approval was obtained from all participating centers. Informed consent was waived due to the retrospective design.

### 2.3 Cohort Definition

Of 203 screened patients, 118 met eligibility criteria and were included in the final analysis. Based on study center, 64 patients from the Affiliated Hospital of Zunyi Medical University, the Second Affiliated Hospital of Guizhou University of Traditional Chinese Medicine, and Guizhou Provincial People's Hospital formed the development cohort; 54 patients from Beijing Hospital and the Myasthenia Gravis Diagnosis and Treatment Center of Shijiazhuang People's Hospital (Hebei Province) formed the external validation cohort.

### 2.4 Evaluation and Collection

Demographic variables included sex, age, and age at onset. Pre‐efgartigimod clinical characteristics included: disease duration; antibody subtype; thymic status; history of thymectomy; disease status at efgartigimod initiation; MGFA classification at efgartigimod initiation; prior treatments (pyridostigmine, prednisone, non‐steroidal immunosuppressants [NSIST], IVIg/plasma exchange); MG‐ADL total score; MG‐ADL bulbar subscore; FVC (absolute value); percent predicted FVC (FVC%); serum IgG titer; and comorbidities. Disease duration was defined as the time from MG onset to first efgartigimod use. The MG‐ADL bulbar subscore assesses speech, chewing, and swallowing. Acute exacerbation was defined as symptom worsening requiring monitoring or treatment. Myasthenic crisis (MC) was defined as rapid respiratory decline necessitating non‐invasive ventilation or endotracheal intubation with mechanical ventilation. Impending myasthenic crisis (IMC) was defined as rapid MG deterioration judged by clinicians to be at risk of MC within days to weeks. Early MSE response was defined as achieving MSE (MG‐ADL ≤ 1) within 4 weeks of starting efgartigimod infusion and maintaining that status for at least 4 weeks. Sensitivity analyses were performed using alternative sustainment windows, including an approximately 2‐week definition (MSE sustained from week 2 to week 4) and an 8‐week definition (MSE sustained from week 4 to week 12), to evaluate the robustness of the model to outcome duration.

In addition, we conducted a comparative analysis using the FDA/EMA‐aligned MG‐ADL responder definition (≥ 2‐point reduction from cycle baseline sustained for ≥ 4 consecutive weeks during the first treatment cycle) to illustrate how predictive performance varies across endpoint definitions.

### 2.5 Statistical Analysis

Analyses were performed in R (version 4.4.3). Two‐sided *p* < 0.05 was considered statistically significant. Continuous variables were assessed for normality using the Shapiro–Wilk test and are presented as mean ± SD (normal) or median (Q1, Q3) (non‐normal). Between‐cohort baseline characteristics were compared using Student's *t*‐test or the Mann–Whitney *U* test, as appropriate. Categorical variables are summarized as *n* (%) and compared using the χ^2^ test or Fisher's exact test. Analyses were conducted as complete‐case analyses; no imputation was performed.

Selection of candidate predictors. Candidate baseline predictors were pre‐specified based on clinical plausibility, published evidence/clinical experience, and availability/standardization across centers. MG‐ADL measures were prioritized to reflect baseline symptom burden relevant to the MSE target; respiratory function (FVC and FVC% predicted) to capture ventilatory involvement and disease severity; and serological markers (serum IgG and antibody status) given the mechanistic relevance of FcRn blockade to pathogenic IgG. Additional demographic and disease‐history variables (e.g., age, disease duration, MGFA class, prior therapies, and comorbidities) were included to account for potential confounding and enhance generalizability.

#### 2.5.1 Model Development and Validation

Baseline factors associated with early MSE response were screened using univariable logistic regression. Variables with *p* < 0.05 in univariable analyses and those deemed clinically important were entered into a multivariable logistic regression model to identify independent predictors. Continuous predictors were modeled on the logit scale with linearity assumed; collinearity was assessed prior to final model specification. A nomogram was constructed based on the final multivariable model. Discrimination was assessed using ROC curves and AUC with 95% confidence intervals (DeLong method). Calibration was evaluated using calibration plots and the Spiegelhalter *Z*‐test (*p* > 0.05 indicating no significant deviation between predicted and observed probabilities). Internal validation was performed using bootstrap resampling (1000 iterations) to estimate optimism‐corrected performance. External validation was conducted by applying the model to the independent validation cohort and reporting AUC and calibration. Clinical utility was evaluated using decision curve analysis comparing net benefit across a range of threshold probabilities against “treat‐all” and “treat‐none” strategies.

#### 2.5.2 Subgroup and Supplementary Analyses

Subgroup validation was performed by diabetes status, overall comorbidity burden, prior IVIg/plasma exchange exposure, and baseline immunosuppressive therapy (Table S1). Discrimination within each subgroup was quantified using AUC with 95% confidence intervals and summarized in a forest plot (Figure S1). For supplementary comparative analyses, we evaluated discrimination under alternative sustained‐MSE duration definitions (2‐week and 8‐week) and compared performance under an FDA/EMA‐aligned MG‐ADL responder endpoint; additionally, we refit a logistic model using the same predictors under that endpoint to benchmark definition‐dependent performance (Table S2).

## 3 Results

### 3.1 Baseline Characteristics

From October 2023 to July 2025, 203 gMG patients across five tertiary centers received efgartigimod 10 mg/kg IV therapy. Ultimately, 118 patients were included—64 in the development cohort and 54 in the validation cohort (Figure 1). The proportions achieving early MSE response were similar across the overall population, development cohort, and validation cohort: 40.68% (48/118), 40.63% (26/64), and 40.74% (22/54), respectively. Baseline demographics and clinical features are summarized in Table 1. The cohorts did not differ significantly in sex, age, age at onset, disease duration, MGFA classification at efgartigimod initiation, MG‐ADL bulbar subscore, or comorbidities. They differed in antibody subtype, disease status at efgartigimod initiation, prior treatments, MG‐ADL total score, absolute FVC, and FVC%.

**TABLE 1:** Baseline demographic and clinical characteristics of the derivation cohort (*n* = 64) and the external validation cohort (*n* = 54).

Variables		Derivation cohort (*n* = 64)	External validation cohort (*n* = 54)	*p*
Gender, *n* (%)	Male	30 (46.9)	23 (42.6)	0.712
	Female	34 (53.1)	31 (57.4)	0.19
Time, median(Q1–Q3)	Age	50 (41–67.3)	63.5 (51.2–69.8)	0.412
	Age at onset	46 (28–61)	58.5 (47.5–67.8)	0.531
	Disease duration	3.5 (0.8–7)	1.1 (0.3–5.9)	0.29
Antibody isotype, *n* (%)	AChR‐Ab	62 (96.9)	52 (96.3)	1
	MuSK‐Ab	4 (6.3)	2 (3.7)	1
	Titin‐Ab	17 (26.6)	3 (5.6)	0.001
	RyR‐Ab	6 (9.4)	1 (1.9)	0.123
Thymic status, *n* (%)	Normal thymus	32 (50.0)	36 (66.7)	0.518
	Thymic hyperplasia	12 (18.7)	7 (12.9)	0.456
	Thymoma	20 (31.2)	11 (20.4)	0.512
	Thymectomy	23 (35.9)	13 (24.1)	0.716
Disease status at efgartigimod initiation, *n* (%)	Stable disease	22 (34.3)	10 (18.5)	0.99
	Acute exacerbation	34 (53.1)	39 (72.2)	0.027
	Impending crisis	5 (7.8)	1 (1.9)	0.034
	Myasthenic crisis	3 (4.7)	4 (7.4)	0.008
MGFA classification at efgartigimod initiation, *n* (%)	IIa	13 (20.3)	10 (18.5)	0.785
	IIb	21 (32.8)	4 (7.4)	0.613
	IIIa	2 (3.1)	10 (18.5)	0.427
	IIIb	18 (28.1)	20 (37.0)	0.731
	IVb	8 (12.5)	2 (3.7)	0.512
	V	2 (3.1)	1 (1.9)	0.689
Prior treatments, *n* (%)	Pyridostigmine	64 (100)	51 (94.4)	0.093
	Prednisone	57 (89)	15 (27.8)	< 0.001
	Tacrolimus	18 (28.2)	10 (18.5)	< 0.001
	Mycophenolate mofetil	18 (28.2)	6 (11.1)	0.008
	IVIg/PE	19 (29.6)	1 (1.9)	< 0.001
	Total MG‐ADL score, median (Q1–Q3)	9.5 (7–12)	6.5 (5–10)	0.007
	Bulbar MG‐ADL subscore, median (Q1–Q3)	2.5 (0.8–4)	2 (0–3)	0.533
	FVC, mean ± SD	1693.3 ± 831.9	1239.1 ± 1137.2	0.039
	FVC%, median(Q1–Q3)	60 (37.4–76)	78.4 (73.2–82.8)	< 0.001
	IgG(g/L), median(Q1–Q3)	12.1 (9.9–14.6)	10.5 (8.7–12.2)	0.081
Comorbidities, *n* (%)	Type 2 diabetes mellitus (T2DM)	9 (14.0)	10 (18.5)	0.845
	Hypertension	15 (23.4)	15 (27.8)	0.672
	Hyperlipidemia	16 (25)	2 (3.7)	0.723
	Osteoporosis	15 (23.4)	2 (3.7)	0.591
	Thyroid disorders	11 (17.1)	2 (3.7)	0.781

*Note:* Continuous variables are presented as Median (Q1–Q3) or Mean ± SD, and categorical variables as *n* (%). Between‐group comparisons used the χ^2^ test or Fisher's exact test for categorical variables and the Mann–Whitney *U* test (or unpaired *t*‐test when appropriate) for continuous variables. Two‐sided *p* < 0.05 was considered statistically significant. AChR‐Ab, acetylcholine receptor antibody; FVC, forced vital capacity; IgG, immunoglobulin G; IVIg, intravenous immunoglobulin; MGFA, Myasthenia Gravis Foundation of America; MuSK‐Ab, muscle‐specific kinase antibody; PE, plasma exchange; Q1–Q3, interquartile range; RyR‐Ab, ryanodine receptor antibody; SD, standard deviation.

### 3.2 Development and Validation of the Nomogram

**FIGURE 2:**
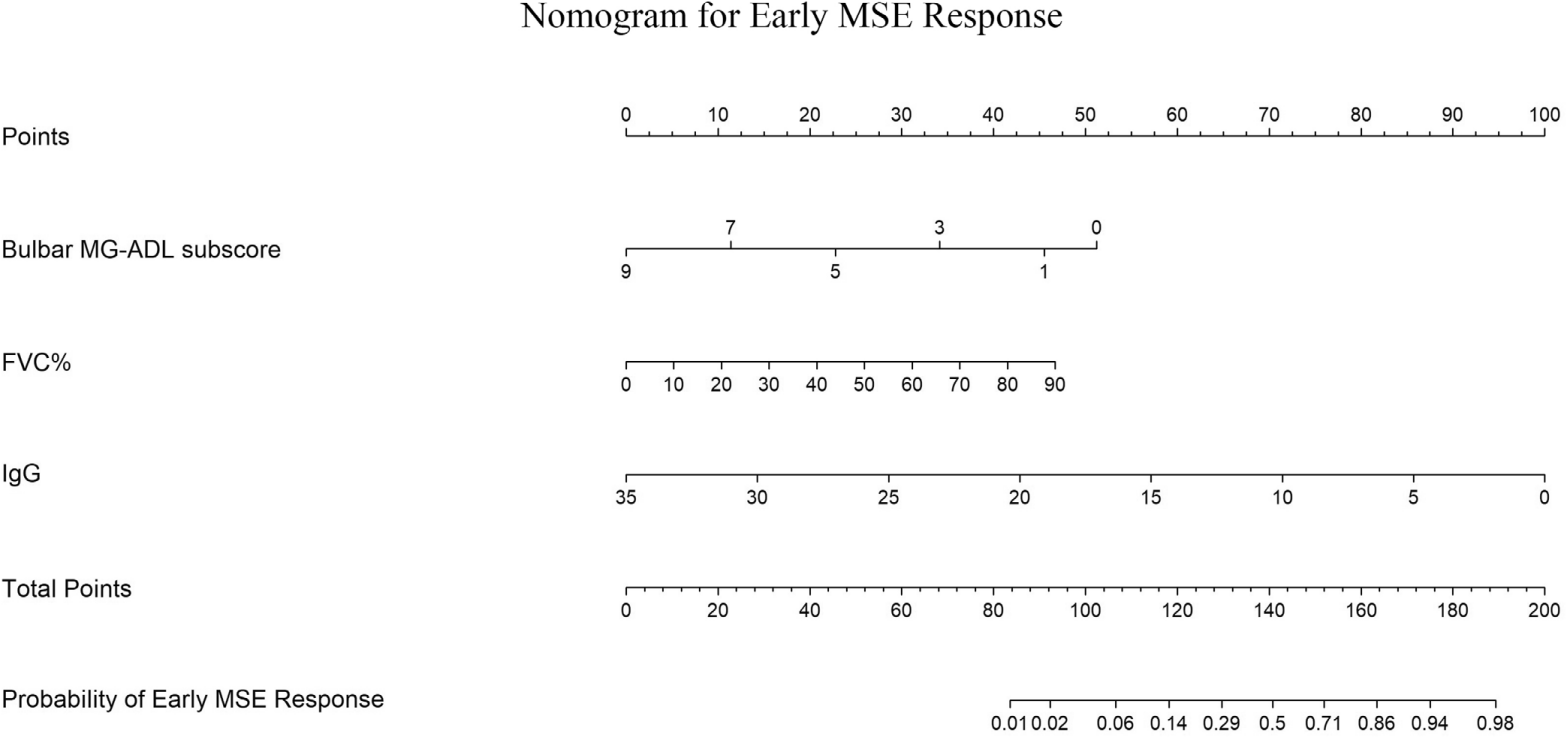
Nomogram for predicting early minimal symptom expression (MSE) response to efgartigimod. The nomogram integrates baseline bulbar MG‐ADL subscore, percent predicted forced vital capacity (FVC%), and serum IgG. For an individual, locate each predictor value on its corresponding axis to assign points, sum the points to obtain the “Total Points,” and project downward to estimate the probability of achieving early MSE response (MG‐ADL ≤ 1 within 4 weeks and sustained ≥ 4 weeks).

Patients were categorized into “MSE response” and “MSE non‐response” groups according to early MSE status. In the development cohort, univariable logistic regression identified significant associations between early MSE response and MG‐ADL total score, MG‐ADL bulbar subscore, FVC (absolute), FVC%, and serum IgG concentration (*p* < 0.05) (Table 2). Variables such as sex, age, disease duration, antibody type, and prior treatments were not significant. Significant variables were then included in multivariable logistic regression, which identified baseline MG‐ADL bulbar score (OR = 0.633, *p* = 0.040), FVC% (OR = 1.042, *p* = 0.048), and IgG level (OR = 0.795, *p* = 0.036) as independent predictors of early MSE response to efgartigimod (Table 2). A nomogram incorporating these predictors was constructed to estimate individual probabilities of early MSE response (Figure 2). Each predictor maps to a point score; summed points yield a total score, which projects to the predicted probability axis.

**TABLE 2:** Univariable and multivariable logistic regression analyses of factors associated with early minimal symptom expression (MSE) response in generalized Myasthenia Gravis (gMG) patients treated with efgartigimod.

Variables		Early MSE responders (*n* = 26)	Early MSE non‐responders (*n* = 38)	Univariate analysis			Multivariate analysis		
				*p*	OR	95% CI	*p*	OR	95% CI
	Female, *n* (%)	14 (53.85)	20 (52.63)	0.924	1.05	0.385–2.880			
	Age, median (Q1–Q3)	52 (38–61)	50 (41–69.75)	0.489	1.01	0.981–1.041			
	Age at onset, median (Q1–Q3)	45 (28–67.5)	46.5 (32.5–59)	0.67	1.006	0.980–1.032			
	Disease duration, median (Q1–Q3)	48 (5.25–84)	36 (24–84)	0.578	1.002	0.996–1.008			
Thymic status, *n* (%)	Normal thymus	13 (50.00)	19 (50.00)	0.996	1	—			
	Thymic hyperplasia	5 (19.23)	7 (18.42)	0.95	1.044	0.271–4.015			
	Thymoma	8 (30.77)	12 (31.58)	0.964	0.974	0.312–3.044			
	Thymectomy, *n* (%)	10 (38.5)	12 (31.6)	0.338	1.648	0.593–4.635			
Disease status at efgartigimod initiation, *n* (%)	Stable disease	10 (38.5)	12 (31.6)	—	1.00	—			
	Acute exacerbation	15 (57.7)	19 (50.0)	0.92	0.95	0.33–2.73			
	Impending crisis	1 (3.8)	4 (10.5)	0.36	0.30	0.03–2.92			
	Myasthenic crisis	0 (0.0)	3 (7.9)	—	—	—			
Disease status at efgartigimod initiation, *n* (%)	II	16 (61.5)	18 (47.4)	—	1.00	—			
	III	9 (34.6)	11 (28.9)	0.88	0.92	0.31–2.73			
	IV	1 (3.8)	7 (18.42)	0.13	0.13	0.01–1.09			
	V	0 (0.0)	2 (5.3)	—	—	—			
Prior treatments, *n* (%)	Pyridostigmine	180 (180–180)	180 (180–240)	0.139	0.994	0.984–1.001			
	Prednisone	40 (30–60)	40 (26.3–60)	0.899	0.999	0.977–1.021			
	Tacrolimus	6 (23.1)	12 (31.6)	0.497	0.834	0.481–1.389			
	Mycophenolate mofetil	10 (38.5)	8 (21.1)	0.574	1.369	0.446–4.234			
	IVIg/PE	4 (15.4)	15 (39.5)	0.045	0.279	0.071–0.906			
	Total MG‐ADL score, median (Q1–Q3)	8 (7–10)	11 (9–13)	**0.01**	0.773	0.626–0.931	0.448	0.904	0.697–1.172
	Bulbar MG‐ADL subscore, median (Q1–Q3)	1 (0–2)	4 (2.3–5)	**< 0.001**	0.477	0.314–0.671	**0.04**	0.633	0.397–0.963
	FVC, Mean ± SD	1961.5 (1623.5–2711.3)	1525 (798.8–2004.5)	**0.004**	1.001	1.000–1.002	0.079	1.001	1.000–1.002
	FVC%, median(Q1–Q3)	75.61 (59.76–81.50)	46.75 (25.08–69.80)	**< 0.001**	1.058	1.028–1.096	**0.048**	1.042	1.002–1.090
	IgG(g/L), median(Q1–Q3)	10.55 (7.62–12.60)	13.08 (11.38–14.89)	**0.005**	0.784	0.650–0.915	**0.036**	0.795	0.628–0.970
Comorbidities, *n* (%)	Type 2 diabetes mellitus (T2DM)	4 (15.3)	5 (13.1)	0.802	1.2	0.271–5.027			
	Hypertension	5 (19.2)	10 (26.3)	0.513	0.667	0.184–2.177			
	Hyperlipidemia	8 (30.77)	8 (21.0)	0.38	1.667	0.527–5.305			
	Osteoporosis	8 (30.77)	9 (23.6)	0.955	0.967	0.285–3.116			
	Thyroid disorders	2 (7.6)	9 (23.6)	0.23	0.42	0.086–1.595			

*Note:* Categorical variables are summarized as *n* (%); continuous variables as median (Q1–Q3) unless otherwise specified as mean ± SD. Univariate *p* values were obtained using χ^2^ or Fisher's exact tests for categorical variables and the Mann–Whitney *U* test (or unpaired *t*‐test when appropriate) for continuous variables. Odds ratios (ORs) with 95% confidence intervals (CIs) were estimated by logistic regression with early MSE response as the outcome. The multivariable model included sex, age, age at onset, disease duration, thymic status, thymectomy, clinical status at efgartigimod initiation, MGFA class (II–V), pyridostigmine dose, prednisone dose, tacrolimus, mycophenolate mofetil, IVIg/PE, Total MG‐ADL and bulbar MG‐ADL scores, FVC (mL), FVC% predicted, and serum IgG. For continuous predictors, ORs are expressed per 1‐unit increase (age/age at onset, years; disease duration, months; pyridostigmine and prednisone, mg/day; FVC, mL; FVC%, % predicted; IgG, g/L; MG‐ADL scores, points). Reference categories: male sex; Normal thymus; no thymectomy; Stable disease (at efgartigimod initiation); MGFA class II; absence of tacrolimus, mycophenolate mofetil, or long‐term IVIg/PE. Two‐sided *p* < 0.05 was considered statistically significant. “—” indicates not estimable due to zero counts. FVC, forced vital capacity; IgG, immunoglobulin G; IVIg, intravenous immunoglobulin; MGFA, Myasthenia Gravis Foundation of America; MG‐ADL, Myasthenia Gravis–Activities of Daily Living; MSE, minimal symptom expression; PE, plasma exchange. Bold values indicates statistical significance.

**FIGURE 3:**
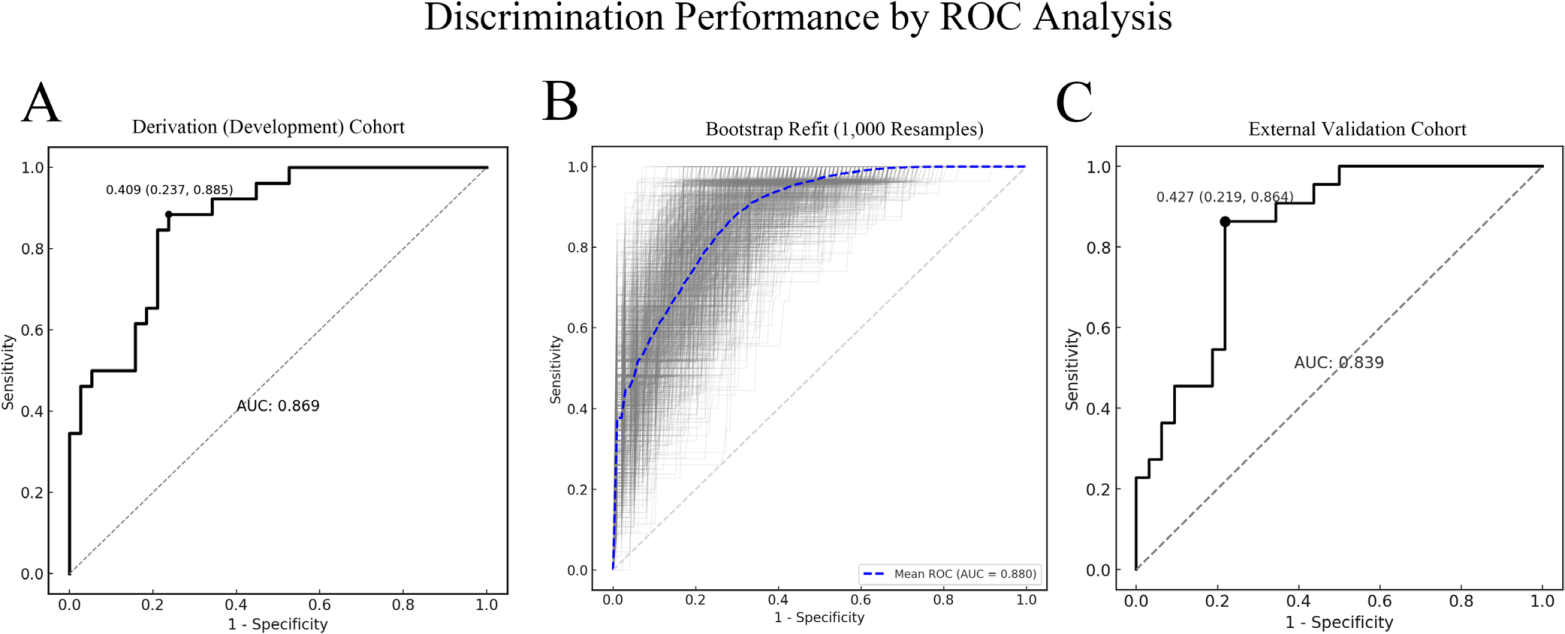
Discrimination of the prediction model by ROC analysis. (A) Derivation cohort (*n* = 64): ROC curve with the optimal operating point indicated (black dot); area under the curve (AUC) = 0.869. (B) Internal validation by bootstrap resampling (1000 refits): Gray lines show ROC curves across resamples; the dashed blue line denotes the mean ROC (AUC = 0.880). (C) External validation cohort (*n* = 54): ROC curve with AUC = 0.839. Diagonal gray line indicates no‐discrimination reference.

**FIGURE 4:**
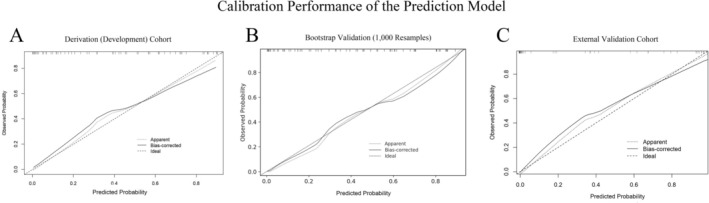
Calibration of the prediction model. (A) Derivation cohort; (B) bootstrap internal validation (1000 resamples); (C) external validation cohort. Dotted line, apparent calibration; solid line, bias‐corrected calibration after bootstrapping; dashed line, ideal 45° line. Tick marks on the top border depict deciles of predicted risk. In all datasets, calibration curves show close agreement with the ideal line; Spiegelhalter *Z*‐tests were non‐significant (derivation *Z* = 1.03, *p* = 0.303; bootstrap *Z* = 0.94, *p* = 0.347; external validation *Z* = 1.17, *p* = 0.242).

**FIGURE 5:**
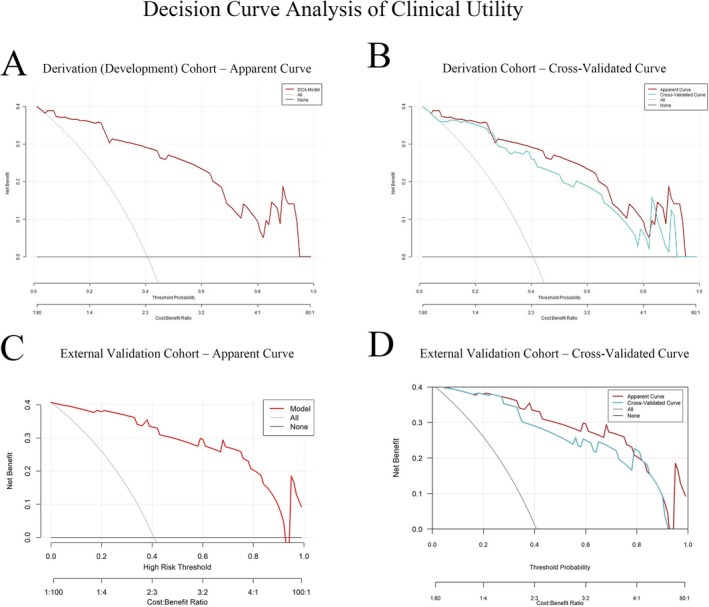
Clinical utility of the nomogram by decision curve analysis (DCA). (A) Derivation cohort: Net benefit of the nomogram compared with “treat‐all” and “treat‐none” strategies across threshold probabilities. (B) Derivation cohort with apparent and cross‐validated DCA curves. (C) External validation cohort: Net benefit curves for the nomogram versus “all” and “none.” (D) External validation cohort with apparent and cross‐validated DCA curves. Across a clinically relevant range of threshold probabilities (approximately 0.05–0.82), using the nomogram to guide efgartigimod initiation (i.e., treat when the predicted probability of early MSE response is ≥ the chosen threshold) confers a higher net benefit than either treating all or treating none. Across a clinically relevant range of threshold probabilities (approximately 0.05–0.82), a nomogram‐guided strategy—initiating efgartigimod when the predicted probability of achieving early MSE response is at or above the selected threshold—yields a higher net benefit than either treating all patients or treating none in both cohorts. Threshold selection may be individualized within this favorable range based on clinical preferences and resource considerations (e.g., a threshold of 0.30). DCA, decision curve analysis; MG‐ADL, Myasthenia Gravis–Activities of Daily Living; MSE, minimal symptom expression.

The nomogram demonstrated strong discrimination in the development cohort: AUC = 0.869 (95% CI: 0.797–0.941) (Figure 3A). The Spiegelhalter Z‐test (*Z* = 1.03, *p* = 0.303) indicated good agreement between predicted and observed outcomes. Calibration (Figure 4A) showed the apparent and bias‐corrected curves closely tracking the ideal 45° line, nearly overlapping at intermediate probabilities (~0.2–0.6) with slight underestimation at the higher‐risk end. Bootstrap internal validation (1000 resamples) yielded an average AUC of 0.880 (Figure 3B), and the bootstrap calibration curve also showed close agreement between predicted probabilities and observed rates (Figure 4B). In the external validation cohort, performance remained robust: AUC = 0.839 (95% CI: 0.760–0.919) (Figure 3C). Calibration analysis showed no significant deviation (Spiegelhalter *Z* = 1.17, *p* = 0.242), with the calibration curve presented in Figure 4C. DCA indicated that, for threshold probabilities from 0.05 to 0.82, model‐guided decisions produced a higher net benefit than “treat‐all” or “treat‐none” strategies (Figure 5A,B). The external validation DCA curves were similar to those of the development cohort, supporting generalizability and potential clinical utility (Figure 5C,D). Decision curve analysis (DCA) demonstrated that using the nomogram to guide efgartigimod initiation provided a higher net benefit than either the “treat‐all” or “treat‐none” strategies across a clinically relevant range of threshold probabilities (approximately 0.05–0.82) in both the derivation and external validation cohorts (Figure 5). This indicates that, within this threshold range, model‐guided decision‐making can improve clinical utility compared with empirical treatment of all patients or no patients. As an example of threshold selection, clinicians may choose a threshold (e.g., 0.30) within this favorable range based on local practice, patient preferences, and resource considerations.

#### 3.2.1 Overall Performance Summary

Overall, the nomogram showed consistent performance across cohorts, with high discrimination in the derivation cohort (AUC = 0.869) and preserved discrimination in external validation (AUC = 0.839). Calibration was acceptable in both cohorts (Spiegelhalter *Z*‐test *p* > 0.05), and decision curve analysis supported clinical utility, showing higher net benefit than “treat‐all” or “treat‐none” strategies across threshold probabilities of approximately 0.05–0.82.

#### 3.2.2 Potential Clinical Implementation

In routine practice, the nomogram can be used at treatment initiation to estimate an individual patient's probability of achieving early MSE response under efgartigimod and to guide baseline counseling and follow‐up planning. For example, patients with lower bulbar symptom burden, higher FVC% predicted, and lower serum IgG would typically receive a higher predicted probability of achieving early MSE response and could be prioritized for efgartigimod with routine monitoring; conversely, patients with prominent bulbar involvement and reduced FVC% predicted may have a lower predicted probability and may warrant closer follow‐up (earlier MG‐ADL reassessment), more conservative counseling regarding expected early benefit, and proactive contingency planning for treatment optimization or escalation if early improvement is not observed. Using a clinically selected threshold informed by DCA (e.g., ≥ 0.30) may facilitate risk‐stratified decision‐making and more efficient allocation of resources for this high‐cost therapy.

Compared with the primary endpoint, discrimination was generally similar under alternative sustained‐MSE duration definitions (with wider confidence intervals for stricter definitions due to fewer events), whereas it decreased when the outcome was redefined using a regulatory‐aligned MG‐ADL responder endpoint. Detailed AUC estimates and 95% CIs are provided in Table S2.

Subgroup discrimination (AUC with 95% CIs) is summarized in Figure S1 and detailed in Table S1, with wider confidence intervals in smaller subgroups.

## 4 Discussion

Efgartigimod's rapid therapeutic effect has been established in ADAPT and ADAPT+ trials, and numerous real‐world studies in China and internationally have further confirmed its efficacy and safety in gMG. A German multicenter retrospective study reported inadequate responses in 20%–49.1% of patients [11]. The high cost of efgartigimod far exceeds commonly accepted cost‐effectiveness thresholds, limiting access among patients with financial constraints [12]. Thus, predicting which patients will benefit early from efgartigimod is essential from both clinical and economic perspectives. Our study addresses this need by providing a nomogram that estimates the probability of an early MSE response, thereby informing individualized treatment decisions.

In this study, efgartigimod was applied as a rapid‐acting therapy for patients—after control of severe infections—who were in acute exacerbation, in impending myasthenic crisis, or otherwise in need of prompt symptom relief, aiming to quickly control inflammation and halt ongoing neuromuscular damage. Our findings indicate that a lower baseline MG‐ADL bulbar score, higher FVC%, and lower serum IgG are clinical predictors of early MSE response to efgartigimod add‐on therapy. The model based on these factors can help identify patients most likely to derive a rapid benefit, supporting early intervention to achieve symptom control and reduce corticosteroid dependence.

The MG‐ADL total score is a validated patient‐reported outcome assessing the impact of MG on daily activities; it is a primary endpoint in MG clinical trials and a rapid, sensitive clinical tool, with higher scores reflecting greater disease activity [13, 14]. In a Chinese real‐world study with a baseline mean MG‐ADL of 7.7 ± 3.6, 69.7% achieved MSE after one efgartigimod cycle [15]. In contrast, in a UK cohort with a baseline mean of 11.2 ± 3.2, only 10.4% achieved MSE by the end of the first cycle, suggesting a greater required magnitude of improvement [5]. Post hoc analyses of ADAPT showed significant improvements across MG‐ADL domains (bulbar, respiratory, limb, ocular), with the greatest reduction in bulbar scores after the first cycle [16]. Multi‐cycle studies reported an 85.88% reduction in bulbar scores after 3 cycles [17]. A German cross‐sectional study found no significant correlation between bulbar symptoms and fatigue severity [17]; research on MG severity measures suggests limb muscle weakness and physical fatigue may signify milder disease, whereas bulbar muscle weakness indicates more severe MG [18]; other prospective studies have also shown a positive correlation between bulbar symptoms and MG severity/activity [19]. In our cohort, 47 patients had MGFA type b involvement and 73% experienced bulbar weakness (speech, chewing, swallowing). One MuSK‐Ab positive patient with severe dysphagia requiring long‐term feeding tube placement did not respond to efgartigimod, whereas those with lower baseline bulbar scores more frequently achieved MSE. This pattern may reflect more extensive antibody‐mediated NMJ injury and longer repair time in severe bulbar involvement, while milder bulbar symptoms may resolve sooner after rapid IgG reduction.

FVC%—comparing an individual's FVC to the predicted value for healthy persons of the same age, sex, height, and weight—is a reliable indicator of respiratory function. In the Quantitative MG (QMG) score, FVC% is categorized as > 80% (normal), 65%–79% (mild reduction), 50%–64% (moderate reduction), and < 50% (severe reduction), underscoring its role in MG severity assessment [20]. Studies show that during myasthenic crisis, mean FVC% is significantly lower (58.9%, 95% CI 37.3–80.6) than in non‐crisis patients (75.9%, 95% CI 74.9–77.0) [20], and refractory MG patients also have lower FVC% than non‐refractory patients (70.1% vs. 74.0%, 95% CI 65.9–74.3 and 72.9–75.1, respectively) [21]. In another study, 77.1% of MGFA class II and 28.6% of class III patients (2/7) had FVC > 80%, whereas none of the class IV patients did, indicating lower FVC% with increasing severity [22]. Preoperative FVC% < 80% was an independent predictor of postoperative myasthenic crisis in 393 thymectomy cases [23]. Although baseline FVC% has not been clearly established as an independent predictor of biologic efficacy, we found higher baseline FVC% associated with increased likelihood of early MSE response to efgartigimod. Notably, FVC% and MGFA class were not independent predictors in a low‐dose rituximab prediction model [24], suggesting FVC% may primarily reflect baseline respiratory severity rather than directly determining biologic responsiveness. Incorporating FVC% into a composite model, however, reminds clinicians of its prognostic relevance and supports individualized care planning.

MG is fundamentally mediated by pathogenic IgG autoantibodies causing NMJ injury. Efgartigimod antagonizes FcRn, lowering total serum IgG and thereby interrupting ongoing NMJ damage. Multiple clinical and real‐world studies have reported correlations between reductions in total serum IgG and improvements in MG‐ADL or QMG scores [25, 26, 27], suggesting efgartigimod efficacy relates to IgG dynamics. Yet clinical evidence linking baseline total IgG to response heterogeneity remains lacking. A Japanese cohort found that the rate of IgG decline predicted symptom improvement after plasma exchange [28]. Although total IgG in MG is often normal to slightly elevated and improvement commonly coincides with reductions in pathogenic IgG—and while MuSK IgG levels correlate with MGFA class in immunotherapy‐treated patients—total IgG is not associated with clinical severity [29]. We observed that lower baseline total IgG was associated with a higher likelihood of early MSE response to efgartigimod. MG patients exhibit elevated levels of all IgG subclasses (IgG1–IgG4) compared with healthy controls, indicating broad immune activation [30]. IgG production depends on inflammatory cytokines and helper T cells; for example, IL‐6 promotes CD4^+^ T‐cell differentiation and B‐cell maturation into IgG‐secreting plasma cells, so total IgG may reflect overall immune activation [31]. Lower baseline total IgG may therefore indicate lower immune activation and faster symptomatic improvement after IgG reduction. Larger prospective studies are needed to validate the relationship between baseline total IgG and efgartigimod outcomes and to evaluate whether quantitative subclass profiling (e.g., pathogenic IgG4) enhances prediction.

Baseline differences between cohorts warrant attention. The development cohort had more severe disease (higher median MG‐ADL total score: 9.5 vs. 6.5, *p* = 0.007) and poorer respiratory reserve (lower FVC%: 60.0% vs. 78.4%, *p* < 0.001). Corticosteroid use was more frequent (89.0% vs. 27.8%), as were IVIg/plasma exchange treatments (29.6% vs. 1.9%). Titin‐antibody positivity was higher (26.6% vs. 5.6%). These findings indicate relatively milder disease and lower immunosuppression burden in the validation cohort. Despite these differences, model discrimination (AUC = 0.839 vs. 0.869) and calibration (Spiegelhalter *p* > 0.05) remained favorable externally, and DCA net benefit profiles were consistent, supporting robustness and generalizability. Nonetheless, model interpretation should consider population context; in milder cohorts, responders may be more readily identified, whereas in more severe or heavily immunosuppressed populations, further validation is warranted.

Limitations: (1) modest sample size; (2) despite external validation, larger, regionally independent validation cohorts are needed to assess broad applicability; (3) retrospective design risks selection bias; MSE defined via the subjective MG‐ADL may be influenced by comorbidities and patient perception; (4) overlapping effects of efgartigimod with conventional immunosuppression could overstate benefit; and (5) development cohort data from resource‐limited western China may limit extrapolation to high‐resource or fully reimbursed settings; (6) COPD‐stratified validation was not feasible because only five patients had COPD in the pooled cohort. In addition, antibody subtype–stratified validation (AChR‐Ab vs. MuSK‐Ab) could not be reliably performed due to the very small number of MuSK‐Ab–positive patients (four in the derivation cohort and three in the external validation cohort, including one double‐positive case).

Future Directions: Larger multicenter prospective validations; incorporation of immunological biomarkers (e.g., IgG subclasses, inflammatory cytokines) and imaging indices to enhance precision; integration of real‐world health‐economic analyses to quantify value; translation of the nomogram into a clinical calculator or mobile application to enable bedside use; and subtype‐specific stratification (e.g., AChR‐Ab‐positive vs. MuSK‐Ab‐positive) to delineate applicability across gMG phenotypes.

## 5 Conclusion

We developed and externally validated an efgartigimod‐specific nomogram based on baseline bulbar MG‐ADL score, FVC% predicted, and serum IgG to estimate the probability of achieving early sustained‐MSE in adult gMG. By quantifying response likelihood at treatment initiation, the model may assist neurologists in individualizing the decision to start efgartigimod, optimizing follow‐up intensity, and improving resource allocation for this high‐cost biologic therapy.

## Author Contributions

Z.L., Z.X. and J.Y. devised the study. T.L. and J.S. carried out the statistical analyses. H.Y., Z.Z., L.Y. and G.Q. coordinated patient recruitment and data acquisition. Y.Y. and H.X. curated the dataset; H.J. and M.Z. verified laboratory aspects. Y.Y. and T.L. wrote the manuscript. All authors reviewed the manuscript. The authors read and approved the final manuscript.

## Funding

This project was supported by the National Natural Science Foundation of China (No. 82360268 and 82471487), the Guizhou epilepsy basic and clinical research scientific and technological innovation talent team project (No. CXTD[2022]013), the Guizhou provincial “hundred‐level” innovative talents funds (No. GCC‐2022‐038‐1), National High Level Hospital ClinicalReaearch Funding, Grant/Award Number: BJ‐2023‐111.

## Ethics Statement

This multicenter study was led by the Department of Neurology, Affiliated Hospital of Zunyi Medical University (lead center). The study protocol and all related documents were approved by the Ethics Committee of the Affiliated Hospital of Zunyi Medical University (Approval No.: KLLY‐2024‐196). Each participating site obtained local ethics approval or formally relied on the lead‐center approval in accordance with national and institutional regulations. Written informed consent was obtained from all participants prior to sample collection. All procedures complied with Good Clinical Practice and the Declaration of Helsinki.

## Consent

The authors have nothing to report.

## Conflicts of Interest

The authors declare no conflicts of interest.

## Supporting information


**Figure S1:** Subgroup validation of the nomogram (pooled cohort). Forest plot showing the discrimination of the nomogram across clinically relevant subgroups defined by diabetes status, overall comorbidity burden, prior IVIg/plasma exchange (PLEX) exposure, and baseline immunosuppressive therapy. Points indicate the AUC and horizontal bars represent 95% confidence intervals. *n* denotes the number of patients in each subgroup, and events denotes the number achieving early MSE. AUCs (95% CIs) were calculated within each subgroup, and estimates in smaller subgroups show wider confidence intervals. AUC, area under the curve; IVIg, intravenous immunoglobulin; MSE, minimal symptom expression; PLEX, plasma exchange; T2DM, type 2 diabetes mellitus.

**Table S1:** Subgroup discrimination of the nomogram in the pooled cohort.

**Table S2:** Discrimination of the nomogram under alternative endpoint definitions.

## Data Availability

The data that support the findings of this study are available on request from the corresponding author. The data are not publicly available due to privacy or ethical restrictions.

## References

[1] J. D. Lunemann , “Getting Specific: Targeting Fc Receptors in Myasthenia Gravis,” Nature Reviews. Neurology 17, no. 10 (2021): 597–598. 10.1038/s41582-021-00547-z 34426685

[2] K. Lazaridis and S. J. Tzartos , “Myasthenia Gravis: Autoantibody Specificities and Their Role in MG Management,” Frontiers in Neurology 11 (2020): 596981. 33329350 10.3389/fneur.2020.596981 PMC7734299

[3] Y. Arora and Y. Li , “Overview of Myasthenia Gravis,” Hospital Practice (1995) 41, no. 4 (2013): 40–50. 10.3810/hp.2013.10.1079 24145588

[4] J. F. Howard , V. Jr. Bril , T. Vu , et al., “Safety, Efficacy, and Tolerability of Efgartigimod in Patients With Generalised Myasthenia Gravis (ADAPT): A Multicentre, Randomised, Placebo‐Controlled, Phase 3 Trial,” Lancet Neurology 20, no. 7 (2021): 526–536. 34146511 10.1016/S1474-4422(21)00159-9

[5] J. Moniz Dionisio , P. Ambrose , G. Burke , et al., “Efgartigimod Efficacy and Safety in Refractory Myasthenia Gravis: UK'S First Real‐World Experience,” Journal of Neurology, Neurosurgery, and Psychiatry 96, no. 4 (2025): 322–328. 39798959 10.1136/jnnp-2024-334086

[6] S. Suzuki , A. Uzawa , Y. Nagane , et al., “Therapeutic Responses to Efgartigimod for Generalized Myasthenia Gravis in Japan,” Neurology Clinical Practice 14, no. 3 (2024): e200276. 38544885 10.1212/CPJ.0000000000200276 PMC10965358

[7] N. Katyal , K. Halldorsdottir , R. Govindarajan , et al., “Safety and Outcomes With Efgartigimod Use for Acetylcholine Receptor‐Positive Generalized Myasthenia Gravis in Clinical Practice,” Muscle & Nerve 68, no. 5 (2023): 762–766. 37695277 10.1002/mus.27974

[8] G. Zhu , H. Zhou , W. Wang , et al., “Application of Efgartigimod in Chinese Patients With Myasthenia Gravis: A Single‐Center Real‐World Prospective Study,” Therapeutic Advances in Neurological Disorders 18 (2025): 17562864241311127. 39839222 10.1177/17562864241311127 PMC11748066

[9] N. J. Silvestri , “Individualized Dosing of Efgartigimod in Patients With Generalized Myasthenia Gravis: Clinical Experience at a Single Center,” Muscle & Nerve 71, no. 3 (2025): 422–428. 39744896 10.1002/mus.28334 PMC11799394

[10] M. Singer , S. Khella , S. Bird , et al., “Single Institution Experience With Efgartigimod in Patients With Myasthenia Gravis: Patient Selection, Dosing Schedules, Treatment Response, and Adverse Events,” Muscle & Nerve 69, no. 1 (2024): 87–92. 37990374 10.1002/mus.28003

[11] N. Huntemann , L. Gerischer , M. Herdick , et al., “C5 Complement Inhibition Versus FcRn Modulation in Generalised Myasthenia Gravis,” Journal of Neurology, Neurosurgery, and Psychiatry 96, no. 4 (2025): 310–321. 39798960 10.1136/jnnp-2024-334404 PMC12015038

[12] P. W. Lien , M. Joshi , J. A. Tice , et al., “Cost‐Effectiveness of Eculizumab and Efgartigimod for the Treatment of Anti‐Acetylcholine Receptor Antibody‐Positive Generalized Myasthenia Gravis,” Journal of Managed Care & Specialty Pharmacy 30, no. 6 (2024): 517–527. 38824625 10.18553/jmcp.2024.30.6.517 PMC11144987

[13] K. Ruzhansky , Y. Li , G. I. Wolfe , et al., “Standardization of Myasthenia Gravis Outcome Measures in Clinical Practice: A Report of the MGFA Task Force,” Muscle & Nerve 72, no. 1 (2025): 56–65. 40260547 10.1002/mus.28417

[14] R. J. Nowak , M. Benatar , E. Ciafaloni , et al., “A Phase 3 Trial of Inebilizumab in Generalized Myasthenia Gravis,” New England Journal of Medicine 392, no. 23 (2025): 2309–2320. 40202593 10.1056/NEJMoa2501561

[15] S. Hao , Z. Ruan , R. Guo , et al., “Efficacy and Safety of Efgartigimod for Patients With Myasthenia Gravis in a Real‐World Cohort of 77 Patients,” CNS Neuroscience & Therapeutics 31, no. 4 (2025): e70391. 40237260 10.1111/cns.70391 PMC12001068

[16] V. Bril , J. F. Howard , C. Jr. Karam , et al., “Effect of Efgartigimod on Muscle Group Subdomains in Participants With Generalized Myasthenia Gravis: Post Hoc Analyses of the Phase 3 Pivotal ADAPT Study,” European Journal of Neurology 31, no. 1 (2024): e16098. 37843174 10.1111/ene.16098 PMC11235734

[17] J. Chen , X. Zhu , H. Zhou , et al., “Efficacy of Multi‐Cycle Efgartigimod in Achieving Minimal Symptom Expression in Myasthenia Gravis: A Comparative Multi‐Center Study,” International Immunopharmacology 154 (2025): 114603. 40186904 10.1016/j.intimp.2025.114603

[18] A. Regnault , T. Morel , C. de la Loge , F. Mazerolle , H. J. Kaminski , and A. A. Habib , “Measuring Overall Severity of Myasthenia Gravis (MG): Evidence for the Added Value of the MG Symptoms PRO,” Neurology and Therapy 12, no. 5 (2023): 1573–1590. 10.1007/s40120-023-00464-x PMC10444722 37166675

[19] J. Chen , S. Li , L. Feng , H. Wang , X. Huang , and H. Feng , “Nomogram for the Acute Exacerbation of Acetylcholine Receptor Antibody‐Positive Generalized Myasthenia Gravis,” Neurological Sciences 44, no. 3 (2023): 1049–1057. 36369308 10.1007/s10072-022-06493-y

[20] N. E. Gilhus , “Myasthenia Gravis, Respiratory Function, and Respiratory Tract Disease,” Journal of Neurology 270, no. 7 (2023): 3329–3340. 37101094 10.1007/s00415-023-11733-y PMC10132430

[21] S. Vohanka , A. Tichopad , M. Horakova , et al., “Burden of Myasthenia Gravis in The Czech Republic: Analysis of the Nationwide Patient Registry,” Neurology and Therapy 14, no. 1 (2025): 227–242. 39630385 10.1007/s40120-024-00682-x PMC11762035

[22] F. Aguirre , R. N. Fernandez , R. M. Arrejoria , et al., “Peak Expiratory Flow and the Single‐Breath Counting Test as Markers of Respiratory Function in Patients With Myasthenia Gravis,” Neurología 12 (2020): 1765. 10.1016/j.nrleng.2020.09.006 35842128

[23] T. Kanai , A. Uzawa , Y. Sato , et al., “A Clinical Predictive Score for Postoperative Myasthenic Crisis,” Annals of Neurology 82, no. 5 (2017): 841–849. 29083502 10.1002/ana.25087

[24] Y. Zhou , R. Guo , X. Xia , et al., “A Predictive Nomogram for Short‐Term Outcomes of Myasthenia Gravis Patients Treated With Low‐Dose Rituximab,” CNS Neuroscience & Therapeutics 30, no. 5 (2024): e14761. 38739094 10.1111/cns.14761 PMC11090079

[25] J. F. Howard , V. Bril , T. M. Burns , et al., “Randomized Phase 2 Study of FcRn Antagonist Efgartigimod in Generalized Myasthenia Gravis,” Neurology 104, no. 3 (2025): e210299. 39813633 10.1212/WNL.0000000000210299 PMC12530329

[26] T. Nomura , M. Imamura , M. Imura , H. Mizutani , and M. Ueda , “Efgartigimod Treatment for Generalized Myasthenia Gravis: A Single‐Center Case Series of 16 Patients,” Frontiers in Neurology 15 (2024): 1472845. 39469071 10.3389/fneur.2024.1472845 PMC11514137

[27] P. Wang , B. Zhang , J. Yin , et al., “Prospective Cohort Study Evaluating Efficacy and Safety of Efgartigimod in Chinese Generalized Myasthenia Gravis Patients,” Frontiers in Neurology 15 (2024): 1407418. 38966082 10.3389/fneur.2024.1407418 PMC11222781

[28] S. Konno and T. Fujioka , “Serum Immunoglobulin G Level Reduction Is a Predictor of Short‐Term Improvement in Patients With Myasthenia Gravis Undergoing Plasmapheresis,” Therapeutic Apheresis and Dialysis 28, no. 1 (2024): 131–140. 37731293 10.1111/1744-9987.14065

[29] I. Koneczny , M. Mane‐Damas , S. Zong , et al., “A Retrospective Multicenter Study on Clinical and Serological Parameters in Patients With MuSK Myasthenia Gravis With and Without General Immunosuppression,” Frontiers in Immunology 15 (2024): 1325171. 38715598 10.3389/fimmu.2024.1325171 PMC11074957

[30] Y. Liu , W. Wang , and J. Li , “Evaluation of Serum IgG Subclass Concentrations in Myasthenia Gravis Patients,” International Journal of Neuroscience 121, no. 10 (2011): 570–574. 21770714 10.3109/00207454.2011.596293

[31] M. Rasel and F. Zahra , Hypergammaglobulinemia (Polyclonal Gammopathy) (StatPearls, 2025). 36256787

