# Supplementary material for: Development and External Validation of a Multivariable Model to Predict Early Minimal Symptom Expression Response in Adult Generalized Myasthenia Gravis Patients Treated With Efgartigimod
Source: CNS Neurosci Ther. 2026 Jan 12;32(1):e70746. doi: 10.1002/cns.70746 (PMC12794272; doi:10.1002/cns.70746)
Supplement: Supplementary file 1 — Figure S1: Subgroup validation of the nomogram (pooled cohort). Forest plot showing the discrimination of the nomogram across clinically relevant subgroups defined by diabetes status, overall comorbidity burden, prior IVIg/plasma exchange (PLEX) exposure, and baseline immunosuppressive therapy. Points indicate the AUC and horizontal bars represent 95% confidence intervals. n denotes the number of patients in each subgroup, and events denotes the number achieving early MSE. AUCs (95% CIs) were calculated within each subgroup, and estimates in smaller subgroups show wider confidence intervals. AUC, area under the curve; IVIg, intravenous immunoglobulin; MSE, minimal symptom expression; PLEX, plasma exchange; T2DM, type 2 diabetes mellitus. [file CNS-32-e70746-s002.docx]

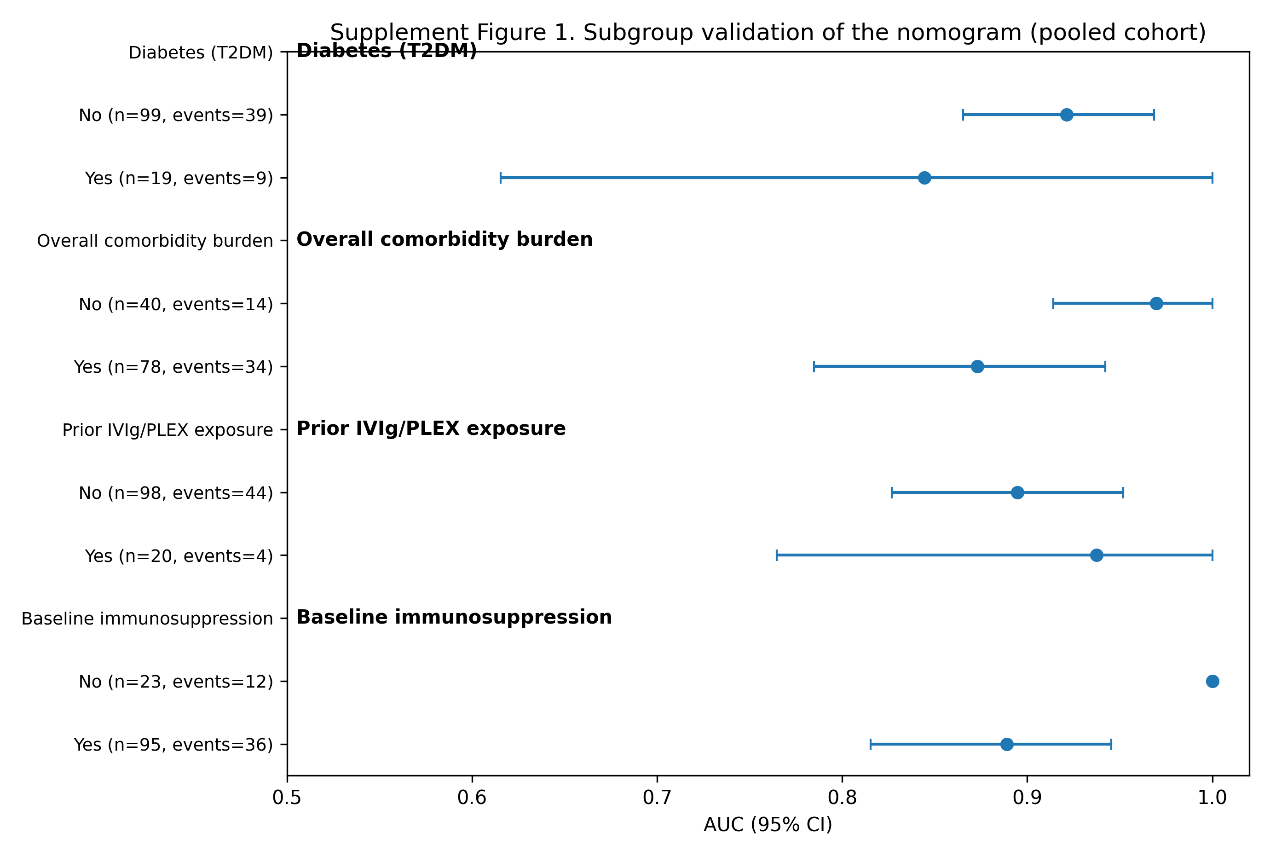


**Supplement Figure 1.** Subgroup validation of the nomogram (pooled cohort).

Forest plot showing the discrimination of the nomogram across clinically relevant subgroups defined by diabetes status, overall comorbidity burden, prior IVIg/plasma exchange (PLEX) exposure, and baseline immunosuppressive therapy. Points indicate the AUC and horizontal bars represent 95% confidence intervals. n denotes the number of patients in each subgroup, and events denotes the number achieving early MSE. AUCs (95% CIs) were calculated within each subgroup, and estimates in smaller subgroups show wider confidence intervals.

Abbreviations: AUC, area under the curve; IVIg, intravenous immunoglobulin; PLEX, plasma exchange; MSE, minimal symptom expression; T2DM, type 2 diabetes mellitus.
