# Supplementary material for: Development and External Validation of a Multivariable Model to Predict Early Minimal Symptom Expression Response in Adult Generalized Myasthenia Gravis Patients Treated With Efgartigimod
Source: CNS Neurosci Ther. 2026 Jan 12;32(1):e70746. doi: 10.1002/cns.70746 (PMC12794272; doi:10.1002/cns.70746)
Supplement: Supplementary file 2 — Table S1: Subgroup discrimination of the nomogram in the pooled cohort. [file CNS-32-e70746-s003.docx]

**Supplementary Table 1.** Subgroup discrimination of the nomogram in the pooled cohort.

| Subgroup | Level | N | Events | AUC (95% CI) |
| --- | --- | --- | --- | --- |
| Diabetes (T2DM) | No | 99 | 39 | 0.921 (0.865–0.969) |
| Diabetes (T2DM) | Yes | 19 | 9 | 0.844 (0.615–1.000) |
| Overall comorbidity burden (≥1 comorbidity) | No | 40 | 14 | 0.970 (0.914–1.000) |
| Overall comorbidity burden (≥1 comorbidity) | Yes | 78 | 34 | 0.873 (0.785–0.942) |
| Prior IVIg/PLEX exposure | No | 98 | 44 | 0.895 (0.827–0.952) |
| Prior IVIg/PLEX exposure | Yes | 20 | 4 | 0.938 (0.765–1.000) |
| Baseline immunosuppression | No | 23 | 12 | 1.000 (1.000–1.000) |
| Baseline immunosuppression | Yes | 95 | 36 | 0.889 (0.815–0.945) |

**Legend**

The table summarizes the nomogram’s discriminative performance within prespecified subgroups defined by diabetes status (T2DM), overall comorbidity burden (≥1 comorbidity), prior IVIg/plasma exchange (PLEX) exposure, and baseline immunosuppressive therapy. For each subgroup level, N denotes the number of patients and Events denotes the number achieving early MSE. Discrimination is reported as the area under the ROC curve (AUC) with 95% confidence intervals.

**Abbreviations:** AUC, area under the curve; ROC, receiver operating characteristic; IVIg, intravenous immunoglobulin; PLEX, plasma exchange; MSE, minimal symptom expression; T2DM, type 2 diabetes mellitus.
