# Supplementary material for: Development and External Validation of a Multivariable Model to Predict Early Minimal Symptom Expression Response in Adult Generalized Myasthenia Gravis Patients Treated With Efgartigimod
Source: CNS Neurosci Ther. 2026 Jan 12;32(1):e70746. doi: 10.1002/cns.70746 (PMC12794272; doi:10.1002/cns.70746)
Supplement: Supplementary file 3 — Table S2: Discrimination of the nomogram under alternative endpoint definitions. [file CNS-32-e70746-s001.docx]

**Supplementary Table S2.** Discrimination of the nomogram under alternative endpoint definitions.

**Legend**

| Endpoint definition | Operational criteria | Derivation events/N | Derivation AUC (95% CI) | External events/N | External AUC (95% CI) |
| --- | --- | --- | --- | --- | --- |
| Alternative definition A: sustained MSE ≥4 weeks | MG-ADL ≤1 at week 4 and week 8 | 26/64 | 0.869 (0.797–0.941) | 22/54 | 0.839 (0.760–0.919) |
| Sensitivity definition B: shorter sustainment (~2 weeks) | MG-ADL ≤1 at weeks 2, 3, and 4 | 3/64 | 0.852 (0.656–1.000) | NA | NA |
| Sensitivity definition C: longer sustainment (≥8 weeks) | MG-ADL ≤1 at weeks 4, 8, and 12 | 8/64 | 0.797 (0.629–0.928) | NA | NA |
| Regulatory-aligned comparison D: MG-ADL responder proxy† | ≥2-point MG-ADL reduction at week 4 with persistence at week 8 | 60/64 | 0.717 (0.333–1.000) | 50/54 | 0.580 (0.340–0.792) |

This table compares the nomogram’s discriminative performance when the outcome was defined using different sustained-MSE duration criteria and a regulatory-aligned MG-ADL responder endpoint. For each definition, the operational criteria used to classify the outcome are listed. Events/N denotes the number of patients meeting the endpoint among the total cohort size, and discrimination is reported as AUC with 95% confidence intervals in the derivation and external validation cohorts. NA indicates that the corresponding endpoint could not be evaluated in the external cohort because the required follow-up assessments (e.g., week 2/3 or week 12 MG-ADL) were unavailable.

Abbreviations: AUC, area under the curve; MG-ADL, Myasthenia Gravis–Activities of Daily Living; MSE, minimal symptom expression.
